# Vasculitis with lichenoid eruptions in Hepatitis B infection – case report

**DOI:** 10.1186/1471-2334-14-S3-P79

**Published:** 2014-05-27

**Authors:** Murugan Sundaram, R Sudha, V Mahalaksmi, S Adikrishnan, M Krishnakanth, M Sundaram

**Affiliations:** 1 Department of Dermatology, Sri Ramachandra Medical College Medical College& Research Institute, Chennai, India; 2 Department of Dermatology, Sri Devi Multispecialty Hospital, Chennai, India

## Background

Hepatitis B virus infection is characterized by asymptomatic to fulminant liver disease, chronic hepatitis, carrier state and liver failure. HBV is known to induce varied skin manifestations, one such presentation as vasculitis with lichenoid eruptions is being reported.

## Case Report

A 50 years old male patient, under treatment for Hepatitis B infection, presented with skin lesions of 4 years duration, starting as erythematous patches slowly becoming thickened and scaly, violaceous papules and patches,. Lesions were moderately itchy, with tenderness and gradual ulceration in the lesions. Lesions involved hands, legs, lower abdomen and trunk. Palms and soles were spared, hair and nails normal. No mucosal involvement. Patient’s hematological and biochemical investigations were within limits. Ultrasound abdomen was normal. ELISA for HIV 1 and 2 were negative. Histopathology of the skin lesion suggestive of lichenoid changes with lymphocytic vasculitis. Patient was managed in conjunction with the gastroenterologist with good improvement.

## Conclusion

Vasculitis with lichenoid eruptions needs to be recognized in the context of Hepatitis B Infection and distinguished from the other manifestations in view of their clinical rarity.

